# Myxobacteria: natural pharmaceutical factories

**DOI:** 10.1186/1475-2859-11-52

**Published:** 2012-04-30

**Authors:** Juana Diez, Javier P Martinez, Jordi Mestres, Florenz Sasse, Ronald Frank, Andreas Meyerhans

**Affiliations:** 1 Molecular Virology Group, Department of Experimental and Health Sciences, Universitat Pompeu Fabra, Barcelona, Spain; 2 ICREA Infection Biology Group, Department of Experimental and Health Sciences, Universitat Pompeu Fabra, Barcelona, Spain; 3 Chemogenomics Laboratory, Research Program on Biomedical Informatics (GRIB), IMIM-Hospital del Mar Research Institute and Universitat Pompeu Fabra, Barcelona, Spain; 4 Department of Chemical Biology, Helmholtz Centre for Infection Research, Braunschweig, Germany

**Keywords:** Myxobacteria, Natural products, Drug discovery, Chemical space

## Abstract

Myxobacteria are amongst the top producers of natural products. The diversity and unique structural properties of their secondary metabolites is what make these social microbes highly attractive for drug discovery. Screening of products derived from these bacteria has revealed a puzzling amount of hits against infectious and non-infectious human diseases. Preying mainly on other bacteria and fungi, why would these ancient hunters manufacture compounds beneficial for us? The answer may be the targeting of shared processes and structural features conserved throughout evolution.

## Commentary

Natural products from plants and microbes have played a pivotal role in drug discovery for more than a century [1-3]. In recent years, myxobacteria have matched fungi, actinomycetes as well as some species of the genus *Bacillus* as top producers of microbial secondary metabolites [4-6]. More importantly, screening campaigns have revealed a large proportion of the myxobacteria secondary metabolism to have activities against human diseases such as cancer, bacterial and viral infections [6-8].

**Figure 1:**
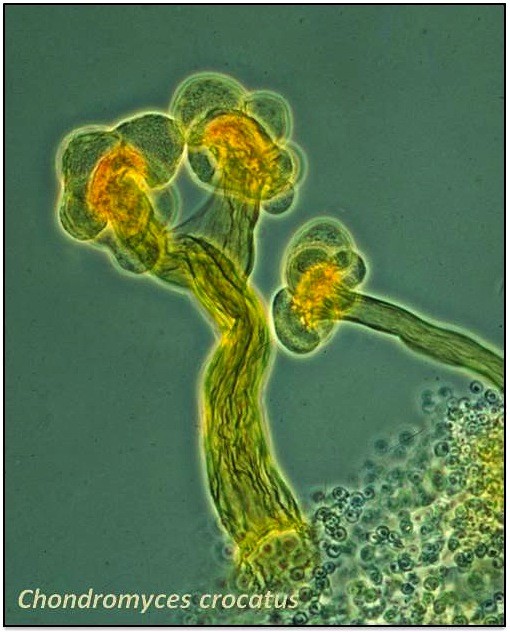
**Image of fruiting bodies from the myxobacterium*Chondromyces crocatus*(courtesy of Hans Reichenbach).**

Myxobacteria are a group of proteobacteria which reside mainly in soil [9,10]. These social microbes move by an axonal cellular motion called gliding [11,12], and although cells grow independently, they form collective swarms to prey and generate transient structures, called fruiting bodies (Figure 1), when resources are scarce [13]. During cooperative feeding, individual cells organize in waves which travel in a rippling-like motion [12,14]. As waves of cells collide, they aggregate in mounds that grow in size forming fruiting bodies that can harbor about 10^5^ individuals. Cells within these structures become myxospores. Sporulation is triggered by signaling at the cell-cell contact surface when nutrients are available, and the myxospores germinate to eventually develop new swarms [11]. To control these processes, myxobacteria have evolved a unique mechanism of extracellular and intracellular signals, including diverse proteins and small metabolites [15].

The chemical space of the myxobacteria metabolome is rare both in diversity and biological activities [5,16,17]. Their secondary metabolites present structural elements not commonly produced by other microbes such as unusual hybrids of polyketides and non-ribosomally made peptides [5,18]. In fact, around 40% of the described myxobacterial compounds represent novel chemical structures [9]. Furthermore, most small molecules from myxobacteria are not glycosylated as opposed to products derived from actinomycetes [19] and they target molecules that are often not targeted by metabolites from other microbes. Examples include inhibitors of mitochondrial respiration and eukaryotic protein synthesis, carboxylase and polymerase inhibitors and molecules that affect microtubule assembly [17]. Although the reasons why myxobacteria display such a large array of secondary metabolites are still not well understood, it has been argued that they confer a competitive advantage in the soil environment and are used to modulate cell-cell interactions within the population [20], to protect ecological niches in their competitive environment [17], and used as weapons for predation [13].

This level of chemical complexity requires an equally complex regulatory network to function, altogether enhancing the survival and competitivity of both the individual and the population [10]. This is reflected in the genetic space employed by myxobacteria for their secondary metabolism. One of the largest bacterial genome reported to date belongs to the myxobacterium *Sorangium cellulosum* with around 20 secondary metabolite loci and probably more to be discovered [15]. Another well studied myxobacterium, *Myxococcus xanthus*, has around 18 secondary metabolite gene clusters accounting for around 9% of its genome [21] which is more than some species of actinomycetes with around 6% of genome coverage for secondary metabolite loci [22,23]. Given this large space on the level of the genome, the known diversity between different myxobacteria and the vast number of different bacterial strains available in various collections, there seems to be an immense room for exploration and exploitation.

The amount of different small molecules from myxobacteria targeting other soil bacteria and fungi, around 29% and 54% respectively, and their higher production rates during exponential growth seems to reinforce the idea of a broad use of secondary metabolites for hunting [13,17]. Any predatory microorganism would benefit greatly from such a diverse armament but why would a large amount of these metabolites be active against human diseases and pathogens? An attractive explanation is that many of these products target shared processes or structural features conserved throughout evolution [24-26]. For example, the LSm1-7 protein complex in mammalian cells was shown to be required for efficient hepatitis C virus (HCV) translation and replication [25]. The Brome mosaic virus (BMV), a plant virus that can replicate in yeast, utilizes the respective yeast homologues for the same processes [27-29]. Likewise, the bacteriophage Q, a plus-strand RNA virus as HCV and BMV, requires Hfq, the homologue of LSm1 in bacteria for its expansion [30]. Thus there is a functional conservation of cellular and viral regulatory elements across kingdoms and virus groups that may be exploited for antiviral drug development. Indeed, a recent screen against processing body proteins that include the LSm1-7 complex revealed several hits from a myxobacterial metabolite library that overlapped with antiviral activities (Martinez et al., unpublished). To learn more about the bioactivity profile of these potent compounds, systematic testing in a broad panel of bioassays as offered by e.g. academic consortia such as EU-OPENSCREEN would be strategically worthwhile. However, to develop a metabolite hit into an applicable pharmaceutical compound is not an easy task, especially given the complexity of their natural product chemistry, side effects and poor bioavailability. Therefore, to make better use of natures pharmaceutical factories, new technologies such as engineering of microorganisms to synthesize complex molecular structures, *in silico* tools to predict the target profile and anticipate potential side effects of those metabolites, and targeted delivery strategies for example via nanoparticles are under the spotlight and will play an increasing role in the future [31-35].

## Competing interests

The authors declare that they have no competing interests.

## References

[B1] Davies J Ryan KS Introducing the parvome: bioactive compounds in the microbial world ACS Chem Biol 2012 7 2 252 259 10.1021/cb200337h 22074935

[B2] Mishra BB Tiwari VK Natural products: an evolving role in future drug discovery Eur J Med Chem 2011 46 10 4769 4807 10.1016/j.ejmech.2011.07.057 21889825

[B3] Newman DJ Cragg GM Natural products as sources of new drugs over the 30 years from 1981 to 2010 J Nat Prod 2012 75 3 311 335 10.1021/np200906s 22316239 PMC3721181

[B4] Arguelles-Arias A Ongena M Halimi B Lara Y Brans A Joris B Fickers P Bacillus amyloliquefaciens GA1 as a source of potent antibiotics and other secondary metabolites for biocontrol of plant pathogens Microb Cell Fact 2009 8 63 10.1186/1475-2859-8-63 19941639 PMC2787494

[B5] Bode HB Muller R Analysis of myxobacterial secondary metabolism goes molecular J Ind Microbiol Biotechnol 2006 33 7 577 588 10.1007/s10295-006-0082-7 16491362

[B6] Weissman KJ Muller R Myxobacterial secondary metabolites: bioactivities and modes-of-action Nat Prod Rep 2010 27 9 1276 1295 10.1039/c001260m 20520915

[B7] Gentzsch J Hinkelmann B Kaderali L Irschik H Jansen R Sasse F Frank R Pietschmann T Hepatitis C virus complete life cycle screen for identification of small molecules with pro- or antiviral activity Antiviral Res 2011 89 2 136 148 10.1016/j.antiviral.2010.12.005 21167208

[B8] Nickeleit I Zender S Sasse F Geffers R Brandes G Sorensen I Steinmetz H Kubicka S Carlomagno T Menche D Argyrin a reveals a critical role for the tumor suppressor protein p27(kip1) in mediating antitumor activities in response to proteasome inhibition Cancer Cell 2008 14 1 23 35 10.1016/j.ccr.2008.05.016 18598941

[B9] Reichenbach H Myxobacteria, producers of novel bioactive substances J Ind Microbiol Biotechnol 2001 27 3 149 156 10.1038/sj.jim.7000025 11780785

[B10] Velicer GJ Vos M Sociobiology of the myxobacteria Annu Rev Microbiol 2009 63 599 623 10.1146/annurev.micro.091208.073158 19575567

[B11] Kaiser D Coupling cell movement to multicellular development in myxobacteria Nat Rev 2003 1 1 45 54 10.1038/nrmicro733 15040179

[B12] Nan B Chen J Neu JC Berry RM Oster G Zusman DR Myxobacteria gliding motility requires cytoskeleton rotation powered by proton motive force Proc Natl Acad Sci USA 2011 108 6 2498 2503 10.1073/pnas.1018556108 21248229 PMC3038734

[B13] Xiao Y Wei X Ebright R Wall D Antibiotic production by myxobacteria plays a role in predation J Bacteriol 2011 193 18 4626 4633 10.1128/JB.05052-11 21764930 PMC3165673

[B14] Berleman JE Kirby JR Deciphering the hunting strategy of a bacterial wolfpack FEMS Microbiol Rev 2009 33 5 942 957 10.1111/j.1574-6976.2009.00185.x 19519767 PMC2774760

[B15] Schneiker S Perlova O Kaiser O Gerth K Alici A Altmeyer MO Bartels D Bekel T Beyer S Bode E Complete genome sequence of the myxobacterium Sorangium cellulosum Nat Biotechnol 2007 25 11 1281 1289 10.1038/nbt1354 17965706

[B16] Bon RS Waldmann H Bioactivity-guided navigation of chemical space Acc Chem Res 2010 43 8 1103 1114 10.1021/ar100014h 20481515

[B17] Weissman KJ Muller R A brief tour of myxobacterial secondary metabolism Bioorg Med Chem 2009 17 6 2121 2136 10.1016/j.bmc.2008.11.025 19109025

[B18] Silakowski B Kunze B Muller R Multiple hybrid polyketide synthase/non-ribosomal peptide synthetase gene clusters in the myxobacterium Stigmatella aurantiaca Gene 2001 275 2 233 240 10.1016/S0378-1119(01)00680-1 11587850

[B19] Rix U Fischer C Remsing LL Rohr J Modification of post-PKS tailoring steps through combinatorial biosynthesis Nat Prod Rep 2002 19 5 542 580 10.1039/b103920m 12430723

[B20] Davies J Spiegelman GB Yim G The world of subinhibitory antibiotic concentrations Curr Opin Microbiol 2006 9 5 445 453 10.1016/j.mib.2006.08.006 16942902

[B21] Bode HB Muller R The impact of bacterial genomics on natural product research Angew Chem Int Ed 2005 44 42 6828 6846 10.1002/anie.200501080 16249991

[B22] Bentley SD Chater KF Cerdeno-Tarraga AM Challis GL Thomson NR James KD Harris DE Quail MA Kieser H Harper D Complete genome sequence of the model actinomycete Streptomyces coelicolor A3(2) Nature 2002 417 6885 141 147 10.1038/417141a 12000953

[B23] Ikeda H Ishikawa J Hanamoto A Shinose M Kikuchi H Shiba T Sakaki Y Hattori M Omura S Complete genome sequence and comparative analysis of the industrial microorganism Streptomyces avermitilis Nat Biotechnol 2003 21 5 526 531 10.1038/nbt820 12692562

[B24] Hong J Role of natural product diversity in chemical biology Curr Opin Chem Biol 2011 15 3 350 354 10.1016/j.cbpa.2011.03.004 21489856 PMC3110584

[B25] Scheller N Mina LB Galao RP Chari A Gimenez-Barcons M Noueiry A Fischer U Meyerhans A Diez J Translation and replication of hepatitis C virus genomic RNA depends on ancient cellular proteins that control mRNA fates Proc Natl Acad Sci USA 2009 106 32 13517 13522 10.1073/pnas.0906413106 19628699 PMC2714764

[B26] Schneider K Kromer JO Wittmann C Alves-Rodrigues I Meyerhans A Diez J Heinzle E Metabolite profiling studies in Saccharomyces cerevisiae: an assisting tool to prioritize host targets for antiviral drug screening Microb Cell Fact 2009 8 12 10.1186/1475-2859-8-12 19183481 PMC2658664

[B27] Diez J Ishikawa M Kaido M Ahlquist P Identification and characterization of a host protein required for efficient template selection in viral RNA replication Proc Natl Acad Sci USA 2000 97 8 3913 3918 10.1073/pnas.080072997 10759565 PMC18116

[B28] Mas A Alves-Rodrigues I Noueiry A Ahlquist P Diez J Host deadenylation-dependent mRNA decapping factors are required for a key step in brome mosaic virus RNA replication J Virol 2006 80 1 246 251 10.1128/JVI.80.1.246-251.2006 16352549 PMC1317526

[B29] Noueiry AO Diez J Falk SP Chen J Ahlquist P Yeast Lsm1p-7p/Pat1p deadenylation-dependent mRNA-decapping factors are required for brome mosaic virus genomic RNA translation Mol Cell Biol 2003 23 12 4094 4106 10.1128/MCB.23.12.4094-4106.2003 12773554 PMC156131

[B30] de Fernandez MT Franze Eoyang L August JT Factor fraction required for the synthesis of bacteriophage Qbeta-RNA Nature 1968 219 5154 588 590 10.1038/219588a0 4874917

[B31] Andexer JN Kendrew SG Nur-e-Alam M Lazos O Foster TA Zimmermann AS Warneck TD Suthar D Coates NJ Koehn FE Biosynthesis of the immunosuppressants FK506, FK520, and rapamycin involves a previously undescribed family of enzymes acting on chorismate Proc Natl Acad Sci USA 2011 108 12 4776 4781 10.1073/pnas.1015773108 21383123 PMC3064383

[B32] Mestres J Seifert SA Oprea TI Linking pharmacology to clinical reports: cyclobenzaprine and its possible association with serotonin syndrome Clin Pharmacol Ther 2011 90 5 662 665 10.1038/clpt.2011.177 21975349 PMC3809033

[B33] Sharma P Garg S Pure drug and polymer based nanotechnologies for the improved solubility, stability, bioavailability and targeting of anti-HIV drugs Adv Drug Deliv Rev 2010 62 45 491 502 19931328 10.1016/j.addr.2009.11.019

[B34] Villaverde A Nanotechnology, bionanotechnology and microbial cell factories Microb Cell Fact 2010 9 53 10.1186/1475-2859-9-53 20602780 PMC2916890

[B35] Zhang MQ Gaisser S Nur EAM Sheehan LS Vousden WA Gaitatzis N Peck G Coates NJ Moss SJ Radzom M Optimizing natural products by biosynthetic engineering: discovery of nonquinone Hsp90 inhibitors J Med Chem 2008 51 18 5494 5497 10.1021/jm8006068 18800759

